# Simultaneous Analysis of Fenpropimorph and Fenpropimorph Acid in Six Different Livestock Products Using a Single-Sample Preparation Method Followed by Liquid Chromatography–Tandem Mass Spectrometry

**DOI:** 10.3390/molecules26195791

**Published:** 2021-09-24

**Authors:** Seon Wook Kim, Da Jung Lim, In Seon Kim

**Affiliations:** Department of Agricultural Chemistry, College of Agriculture and Life Sciences, Chonnam National University, Gwangju 61186, Korea; 1tjsdnr1@gmail.com (S.W.K.); limdajung87@gmail.com (D.J.L.)

**Keywords:** fenpropimorph, livestock products, pesticide, QuEChERS, LC-MS/MS

## Abstract

Pesticides in livestock products must be measured to ensure food safety. We developed a single-sample preparation method followed by liquid chromatography–tandem mass spectrometry (LC-MS/MS) for simultaneous determination of fenpropimorph and fenpropimorph acid in six different livestock products. The extraction method was a modification of the quick, easy, cheap, effective, rugged, and safe (QuEChERS) method and was validated according to the CODEX guidelines. The matrix-matched calibration curves for fenpropimorph and fenpropimorph acid exhibited good linearity, with coefficients of determination (R^2^ values) higher than 0.998. The limit of detection (LOD) and the limit of quantitation (LOQ) were 1.25 and 5.0 µg kg^−1^, respectively. The average recovery values ranged from 61.5% to 97.1% for samples fortified to the LOQ, 2 × LOQ, and 10 × LOQ. The method fully complied with the CODEX guidelines and was successfully applied to real samples obtained from domestic markets.

## 1. Introduction

The total consumption of livestock products is rising as the human population and personal incomes increase [1]. For example, the global consumption of meat (in million metric tons) has increased steadily and rapidly since 1961, reaching approximately 340 million tons in 2018 [2]. Livestock is exposed to pesticides on consumption of animal feeds made from agricultural by-products, such as cereal grains and straw [3,4,5]. Therefore, greater consumption of livestock products leads to greater exposure to pesticides. Thus, analytical methods for monitoring pesticide residues in livestock products are required to ensure food safety.

Livestock products, such as meat, milk, and eggs, are complex matrices containing fatty materials that may interfere with pesticide extraction. Methods that eliminate interfering materials are critical to improve the efficiency of analytical tests of pesticide products in livestock [6,7]. Typical cleanup methods for fatty samples include matrix solid-phase dispersion, freezing and centrifugation, solvent–solvent partitioning, and solid-phase extraction (SPE) [8,9]. The quick, easy, cheap, effective, rugged, and safe (QuEChERS) method is a powerful tool for sample preparation and cleanup of agricultural and animal product matrices prior to instrumental analysis of pesticides [10,11,12].

Fenpropimorph, *cis*-4-[(*RS*)-3-(4-*tert*-butylphenyl)-2-methylpropyl]-2,6-dimethylmorpholine, is a fungicide exhibiting systemic activity against the fungal diseases of various crops, such as grains and vegetables [13]. Although fenpropimorph is primarily used for fungal control, unexpected adverse effects of morpholines on sterol biosynthesis have been reported in mammals and higher plants [14,15,16]. These studies thus suggest that residues of fenpropimorph in edible animal and plant products should be monitored continuously to ensure public safety. Fenpropimorph is an enantioenriched chiral chemical that is stereoselectively metabolized by plants [17,18]. Although fenpropimorph can usually be metabolized to low-toxicity compounds in plants and livestock [19], fenpropimorph acid, a typical metabolite, is included in the list of residues important for monitoring and risk assessment [20]. Thus, evaluation of fenpropimorph residues in livestock products requires an analytical technique that simultaneously determines fenpropimorph and fenpropimorph acid levels.

A number of methods for determination of fenpropimorph in agricultural and insect products have been reported. Gas chromatography–tandem mass spectrometry (GC-MS/MS) has been used after application of modified QuEChERS methods to determine fenpropimorph in agricultural samples [21,22]; a buffered organic solvent was used for sample extraction. A GC-MS/MS method was also used for multi-residue analyses of pesticides (including fenpropimorph) in animal feed samples [23]; organic solvent partitioning and SPE were used for sample preparation. A simpler method of sample preparation (followed by liquid chromatography–tandem mass spectrometry (LC-MS/MS)) was used for multi-residue screening of pesticides (including fenpropimorph) in fruit and vegetable samples; the samples were frozen at −20 °C for 24 h and then subjected to cryogenic milling in dry ice [24]. More recently, LC-MS/MS analyses coupled with modified QuEChERS methods have been used to complement GC/MS when performing multi-residue analyses of pesticides (including fenpropimorph) in insect and plant samples [25,26,27].

The various methods for fenpropimorph determination were mainly derived based on agricultural samples; few studies employed animal products. Additionally, simultaneous determination of fenpropimorph and fenpropimorph acid in livestock products has not been described. Korea has several pesticide monitoring programs, operated by the Ministry of Food and Drug Safety (MFDS), for domestic and imported livestock products, but the MFDS does not simultaneously measure fenpropimorph and its acid residues. A method of fenpropimorph residue analysis should also be capable of detecting fenpropimorph acid residues because the acid is now included in the list of materials to be monitored.

In this study, we developed an analytical method for simultaneous determination of fenpropimorph and fenpropimorph acid in livestock products using LC-MS/MS. The samples were extracted and cleaned using a modified QuEChERS method to process six different livestock samples prior to LC-MS/MS analysis. The method was validated according to the CODEX guidelines. Inter-institutional validation was also conducted.

## 2. Results and Discussion

### 2.1. Instrumental Conditions

The instrumental conditions were optimized in terms of the mobile phase, analytical column, mass ions, and instrumental parameters. The mobile phase was selected to ensure well-defined peaks of fenpropimorph and fenpropimorph acid when the solvent changed. A mixture of methanol and water with 0.1% (*v*/*v*) formic acid was found to be appropriate, as in previous studies [26]. Higher concentrations of formic acid did not significantly improve the peak shapes. A C18 column is commonly used for the chromatographic separation of fenpropimorph via LC-MS/MS from structurally similar chiral fungicides [17] and 155 pesticides [27] in agricultural samples. We successfully separated fenpropimorph and fenpropimorph acid on a C18 column, suggesting that a C18 column can be applicable to the separation of the analytes in both agricultural and livestock samples. 

The mass ions of fenpropimorph and fenpropimorph acid are presented in Table 1. The most abundant mass ions were determined by direct injection of standard solutions into the MS, as *m/z* 304.4, *m/z* 147.2, *m/z* 132.1, and *m/z* 117.2 for fenpropimorph and *m/z* 334.3, *m/z* 117.1, *m/z* 107.1, *m/z* 105.0, and *m/z* 98.3 for fenpropimorph acid; these were the major fragment ions. Of these, the mass ions *m/z* 304.4, *m/z* 147.2, and *m/z* 132.1 for fenpropimorph and *m/z* 334.3, *m/z* 107.1, and *m/z* 98.3 for fenpropimorph acid were the most abundant. Thus, the selected mass ions were used for quantitative and qualitative determination of the target analytes.

The instrumental parameters were optimized to ensure high-quality MS chromatograms; we adjusted the source and desolvation temperatures, and the desolvation, cone, and collision gas flows. The optimum conditions were 150 °C, 400 °C, 650 L h^−1^, 50 L h^−1^, and 0.3 mL min^−1^, respectively. The collision energy values for the quantitative and qualitative ions were 32 and 45 eV, respectively, for fenpropimorph and 36 and 38 eV, respectively, for fenpropimorph acid.

### 2.2. Sample Extraction and Cleanup Methods

The sample preparation procedure was a modification of a QuEChERS-based method recommended by the MFDS that has been used to determine fenpropimorph in agricultural products [28]. The MFDS method is not applicable for simultaneous analysis of the fenpropimorph and fenpropimorph acid in livestock samples. Thus, we aimed to develop a simultaneous method for determination of the analytes in livestock. For this, the method was modified in terms of the extraction solvent and cleanup procedure and met the criteria of the SANTE guidelines [29]. Recovery tests were performed using milk and egg samples spiked to 5 µg kg^−1^ (limit of quantitation (LOQ), 10 µg kg^−1^ (2 × LOQ), and 50 µg kg^−1^ (10 × LOQ)). We used milk and egg typically to optimize sample preparation in the recovery tests, because they may have complex matrices that interfere with pesticide extraction, as reported previously [30]. We considered that if the recoveries from milk and egg met the SANTE criteria, those from the other four matrices would also be reproducible and reliable. For sample cleanup, a dispersive-SPE (d-SPE) tube containing MgSO_4_ (25 mg), PSA (25 mg), and C18 (25 mg) was used, as previously reported. However, the extraction method required modification to improve the recovery of both analytes. We thus added acetic acid to the extraction solvent (acetonitrile) at ratios of 1.0, 3.0, and 5.0% (*v*/*v*). 

**Figure 1:**
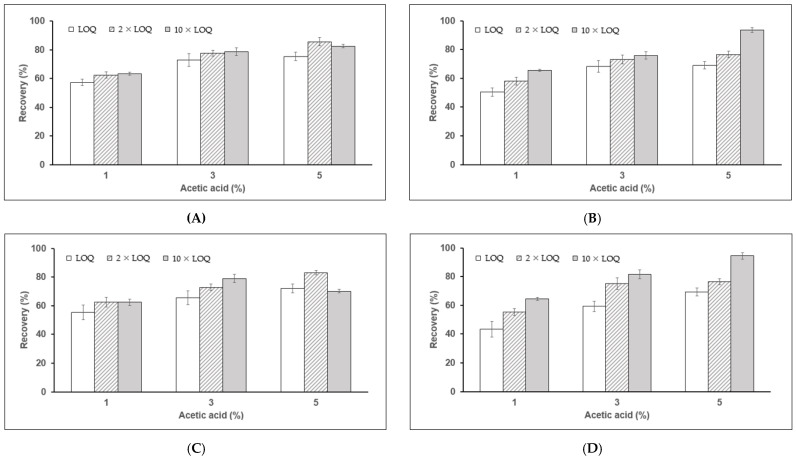
Effects of acetic acid concentration on the recoveries of fenpropimorph (**A**,**B**) and fenpropimorph acid (**C**,**D**) fortified in the milk (**A**,**C**) and egg (**B**,**D**) samples. Data are means ± SD of 5 replicates.

The recoveries of fenpropimorph and fenpropimorph acid are shown in Figure 1. The recoveries of fenpropimorph from milk were 57.4–63.4%, 72.9–78.8%, and 75.5–85.7% when the extraction solvent contained 1.0, 3.0, and 5.0% (*v*/*v*) acetic acid, respectively, and were 50.4–65.6%, 68.2–76.0%, and 75.5–85.7% for the egg samples. The recoveries of fenpropimorph acid from milk were 55.3–62.6%, 65.5–79.0%, and 70.1–83.1% when the extraction solvent contained 1.0, 3.0, and 5.0% (*v*/*v*) acetic acid, respectively, and were 43.3–64.46%, 59.4–81.6%, and 69.3–94.5% from the egg samples. All relative standard deviations (RSDs) were less than 10%, as required by the SANTE guidelines [29]. We found that 5.0% (*v*/*v*) acetic acid optimizes sample extraction. Thus, 5.0% (*v*/*v*) acetic acid in acetonitrile was used for sample extraction throughout the study.

### 2.3. Method Validation

The method established above was validated following the CODEX guidelines [31]. Method validation was based on the linearity of standard calibration, sensitivity, matrix effects, and the accuracy and precision of the target analyte data. The matrix calibrations of fenpropimorph and fenpropimorph acid for various livestock matrices were evaluated from 1.25 to 50 µg L^−1^. The coefficients of determination (R^2^ values) were 0.998–0.999 for fenpropimorph and fenpropimorph acid in all matrices (Table 2), indicating good calibration linearity of the analytes. The limit of quantitation (LOQ) was 0.005 mg kg^−1^ for all analytes, indicating high sensitivity for the determination of fenpropimorph and fenpropimorph acid.

The matrix effects were −0.71–3.17% for fenpropimorph and 1.17–3.17% for fenpropimorph acid in different matrices (Table 3). This suggests insignificant matrix interference with the single-sample preparation method examined in our study. The matrix effect can be ignored if the absolute value of the effect is less than 10% [32]. Thus, the method developed in our study would not have any erroneous quantitation due to the interferences of matrices with the extraction of the target analytes.

Accuracy and precision were investigated by performing recovery tests of fenpropimorph and fenpropimorph acid spiked into livestock samples to the LOQ, 2 × LOQ, and 10 × LOQ. The recoveries are listed in Table 4. The results could demonstrate that the method is accurate and precise. The recoveries from milk and egg were lower than those from other samples, probably attributable to interference by the milk and egg matrices with extraction of the target analytes, as suggested previously in other studies [33]. The recoveries at the LOQ level ranged from 69.1% to 92.4% for fenpropimorph and 69.3% to 111.1% for fenpropimorph acid. Recoveries higher than 70% were observed at the 2 × LOQ and 10 × LOQ levels (range: 70.1–111.2%). The coefficient of variation (CV) of recovery, based on the RSD, was less than 10% for all samples. These data fully meet the CODEX criteria (CAC/GL 71-2009), suggesting that the method is accurate and precise.

**Figure 2:**
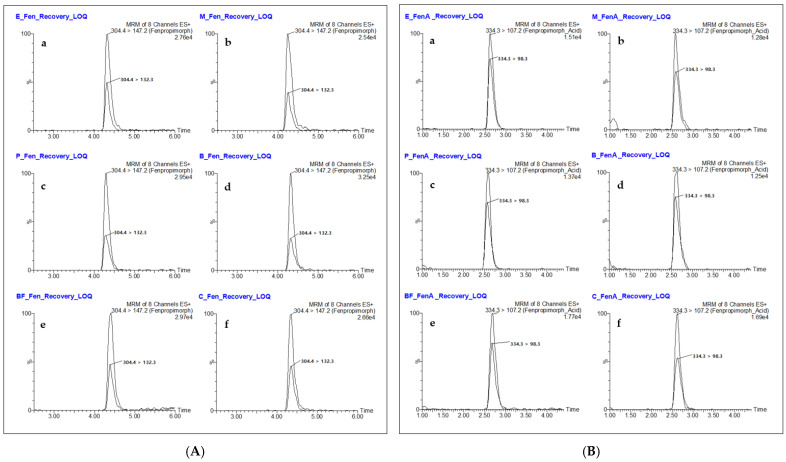
Typical LC-MS/MS chromatograms of two transitions of fenpropimorph (**A**) and fenpropimorph acid (**B**) fortified at the LOQ level in the egg (**a**), milk (**b**), pork bacon (**c**), beef steak (**d**), beef fat (**e**), and chicken leg meat (**f**) samples.

Subsequent LC-MS/MS analysis clearly revealed fenpropimorph and fenpropimorph acid at the lowest levels tested, as shown in Figure 2, as confirmed by the ion ratios of two multiple reaction monitoring (MRM) transitions in the standard solutions and samples. For fenpropimorph, the ion ratios of the samples were in the range of 0.34–0.45, close to the ion ratios of 0.36–0.48 in the standard solutions. For fenpropimorph acid, the ion ratios ranged from 0.50 to 0.78 in the standard solutions and 0.55 to 0.75 in the samples; thus, they were also similar. From these data, the relative differences in ion ratios were calculated as −12.42–3.64% for fenpropimorph and –13.38–7.47% for fenpropimorph acid (Table 5). According to the SANTE/11813/2017 document of the European Commission [34], which validates analytical quality control methods, the sample ion ratio must be within ±30% of the average of the standard calibration value. Our method meets this criterion, so it can really confirm the presence of fenpropimorph and fenpropimorph acid in livestock samples.

Livestock samples, such as milk, egg, and meat, contain complex matrices interfering with pesticide extraction. Lipids, peptides, carbohydrates, proteins, and amines can be found as endogenous substances in the samples [35]. These substances are one of primary factors that may affect the analytical efficiency of pesticides in livestock samples, generating a suppression and enhancement of the matrix effect. More severe suppression of the matrix effect was observed in livestock samples spiked with pesticides at a low level [36], suggesting that the endogenous materials may decrease significantly the signal sensitivity of the analytes at a low level. To increase the signal sensitivity of the targeted analytes in animal-derived samples, different cleanup solid phases should be employed depending on different matrix interferences for sample dispersion [37]. The method developed in our study used a single-sample preparation method for six different sample matrices. The method exhibited low suppression and enhancement of the matrix effect of less than 15%, indicating that the sample cleanup was successful to meet the SANTE guidelines. These observations demonstrated that any erroneous analysis due to matrix interference was avoidable in the established method.

### 2.4. Inter-Institutional Validation of the Established Method

The method was subjected to inter-institutional validation by the Eco-Friendly Agricultural and Biological Research Center (EABRC), a Korean institute certified for pesticide residue analysis. EABRC recovered fenpropimorph and fenpropimorph acid from the livestock samples, as described above; the results are presented in Table 6. Fenpropimorph recovery ranged from 63.9% to 86.2% and fenpropimorph acid recovery from 65.1% to 95.9%. The RSD values were all below 10%. The recovery values are similar to the values presented in Table 4 and meet the CODEX criteria, demonstrating that the method is reproducible. Thus, our method can be used by institutions for simultaneous determination of fenpropimorph and fenpropimorph acid in livestock products. 

### 2.5. Application of the Established Method

**Figure 3:**
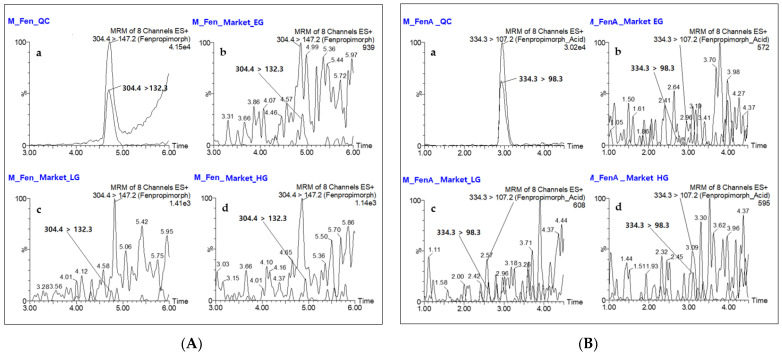
Typical LC-MS/MS chromatograms of two transitions of fenpropimorph (**A**) and fenpropimorph acid (**B**) in real milk samples: (**a**) samples spiked at 0.01 µg/kg; (**b**–**d**) samples from domestic markets. The milk samples from three markets are presented as typical examples of real samples.

The method was used to simultaneously determine fenpropimorph and fenpropimorph acid in real livestock products purchased from domestic markets in Gwangju and Suncheon, Republic of Korea (n = 4). The livestock samples were prepared as described above and subjected to LC-MS/MS analysis. No residue was detected in any sample, as shown in Figure 3.

The residues in real samples were further confirmed based on the relative difference of ion ratios. The relative difference of ion ratios ranged from −7.15 to 4.81% for the spiked samples at the 2 × LOQ, while the difference was in the range of −98.33–2382.63% for real samples (Table 7). The relative difference of ion ratios in real samples exceeded significantly the tolerance limits, ±30%, recommended by the SANTE guidelines. This revealed that the presence of fenpropimorph and fenpropimorph acid in real samples was not confirmed really. If the targeted analytes were presented in real samples, the relative difference in ion ratios would be similar to the spiked samples. Overall, the developed method can monitor simultaneously fenpropimorph and fenpropimorph acid in livestock products.

## 3. Materials and Methods

### 3.1. Chemicals and Reagents

Analytical standards of fenpropimorph (96.2%) and fenpropimorph acid (99.7%) were purchased from Sigma-Aldrich (St. Louis, MO, USA) and LGC Standards (Teddington, Middlesex, UK), respectively. All organic solvents used in this study were of HPLC grade, purchased from J.T Baker (Phillipsburg, NJ, USA). Other chemicals were of analytical grade, purchased from Junsel Chemical Co. (Chuo-ku, Tokyo, Japan). QuEChERS kits were obtained from Agilent Technologies (Santa Clara, CA, USA).

### 3.2. Sample Preparation

The methods for sample extraction and cleanup were developed by modifying a QuEChERS method recommended by the Ministry of Food and Drug Safety, Korea. Milk and egg samples were used as typical samples for establishment of the method. For sample extraction, livestock samples (1 kg) were homogenized thoroughly with dry ice and a portion (5 g) of the samples was transferred into a centrifuge tube (50 mL) containing 0.5 mL of ascorbate buffer solution and acetic acid in acetonitrile (10 mL). The ascorbate buffer solution was prepared by dissolving ascorbic acid (7.5 g) and sodium ascorbate (7.5 g) in water (100 mL). Acetic acid was used at ratios of 1.0–5.0% (*v*/*v*) in acetonitrile. The sample mixture was vortexed vigorously for 2 min, and anhydrous MgSO_4_ (4 g), sodium acetate (1 g), sodium citrate dehydrate (1.0 g) and sodium citrate dibasic sesquihydrate (1 g) were added, followed by shaking vigorously for 2 min. The mixture was then centrifuged at 3500 rpm for 5 min, and the supernatant (15 mL) was placed at −20 °C for 30 min, followed by centrifugation at 3500 rpm for 5 min. An aliquot (1.0 mL) was used for sample cleanup. The sample cleanup was performed using a QuEChERS kit consisting of anhydrous MgSO_4_ (150 mg), PSA (25 mg), and C18 (25 mg) in a dispersive SPE tube. The sample (1.0 mL) was mixed thoroughly with the kit for 1 min and centrifuged at 8000 rpm for 3 min. An aliquot (0.5 mL) of the supernatant was mixed with formate buffer solution (0.4 mL) and methanol (0.1 mL). The formate buffer solution was prepared by dissolving ammonium formate (100 mM) and formic acid (0.1%, *v*/*v*) in water. The sample solution was finally filtered through a fiber membrane filter (0.2 µm, PTFE-H) prior to LC-MS/MS analysis.

### 3.3. Method Validation

The method validation was conducted in terms of calibration linearity, sensitivity, accuracy, precision, and the matrix effect, according to the CODEX guidelines [31]. Matrix-matched standard calibrations were performed for quantitative determination of fenpropimorph and fenpropimorph acid. The calibration solutions were prepared by adding blank matrix solutions to standard working solutions as follows: 0.5 mL of blank matrix solution, 0.4 mL of buffer solution, and 0.1 mL of standard working solution with six different concentrations in the range of 12.5 to 500 µg L^−1^. The buffer solution consisted of ammonium formate (100 mM) and formic acid (0.1%, *v*/*v*) in water. The method sensitivity was investigated based on the limit of quantitation (LOQ) value. The LOQ was determined at a signal-to-noise (S/N) ratio of 10:1 and calculated as follows: LOQ (mg kg^−1^) = (minimum detectable amount (ng) of analytes/sample injection volume (µL)) × (final sample volume (mL)/sample amount (g)). The accuracy and precision of the method were evaluated by the recovery tests. The recovery tests were performed in five replicates at levels of the LOQ, 2 × LOQ, and 10 × LOQ by investigating the concentration detected and fortified in the livestock samples. The coefficient of variation (CV) of the recovery tests was calculated by considering the average recovery and standard deviation, as previously described [38]. The matrix effect (ME) of the sample matrices on the standard calibration was calculated as follows [39,40]: ME (%) = [(slope of calibration curve in matrix-matched standard slope of calibration curve in solvent standard)/(slope of calibration curve in solvent standard)] × 100. 

The developed method was subjected to inter-institutional validation by an official institute of the government of Korea. The inter-institutional validation of the method was performed to examine whether the method could be used as an official method for simultaneous determination of fenpropimorph and fenpropimorph acid in livestock products. For this, the developed method was examined by EABRC.

### 3.4. Instruments

A Waters model Xevo TQD-MS triple quadrupole MS/MS spectrometer was used for simultaneous determination of fenpropimorph and fenpropimorph acid in the livestock samples. The MS/MS spectrometer was equipped with a Waters model ACQUITY^TM^ UPLC system. The analytical column was a CAPCELL CORE C18 stainless column (Osaka Soda, 150 × 2.1 mm, 2.7 μm thickness). The mobile phase consisted of methanol and water containing 0.1% (*v*/*v*) formic acid, and it was flowed at 0.4 mL min^−1^ as follows: 30% methanol in isocratic conditions for 0.5 min, 60% methanol with a linear gradient for 3.0 min, 80% methanol with a linear gradient for 2.0 min, 80% methanol in isocratic conditions for 1.0 min. The electron spray ionization (ESI) method in positive ion mode was used for the MS/MS analysis. The LC-MS/MS conditions were optimized by adjusting instrumental parameters to obtain good resolution of the target ions.

## 4. Conclusions

The method described in this study was developed by combining a modified QuEChERS method with LC-MS/MS. The method was validated in terms of calibration linearity, accuracy, precision, sensitivity, and selectivity. The developed method met the criteria of the CODEX guidelines and proved highly suitable for simultaneous determination of fenpropimorph and fenpropimorph acid in livestock products. The method was also subjected to inter-institutional validation to determine whether it could be widely used in Korea. The developed method was fully validated for simultaneous determination of fenpropimorph and fenpropimorph acid residues in real samples of livestock products obtained from domestic markets.

## Figures and Tables

**Table 1:** Instrumental conditions for analysis of fenpropimorph and fenpropimorph acid in multiple reaction mode.

Chemical	Precursor Ion (*m/z*)	Product Ion (*m*/*z*)	
		Quantitative	Qualitative
Fenpropimorph	304.4	147.2	132.3
Fenpropimorph acid	334.3	107.2	98.3

**Table 2:** Linearity of matrix-matched standard calibration and limits of quantitation (LOQs) of the target analytes.

Analyte	Coefficient Values of Determination in the Different Matrices (R^2^)						LOQ (mg kg^−1^)
	Egg	Milk	Pork Bacon	Beef Steak	Beef Fat	Chicken Leg Meat	
Fenpropimorph	0.999	0.999	0.999	0.998	0.998	0.999	0.005
Fenpropimorph acid	0.998	0.998	0.998	0.998	0.999	0.999	0.005

**Table 3:** Matrix effects of the livestock matrices on the calibration of the target chemicals.

Chemical	Matrix Effect (%)					
	Egg	Milk	Pork Bacon	Beef Steak	Beef Fat	Chicken Leg Meat
Fenpropimorph	2.84	3.17	−2.52	1.44	−0.71	1.53
Fenpropimorph acid	3.17	2.62	2.63	1.17	2.42	2.92

**Table 4:** Recovery values of fenpropimorph and fenpropimorph acid fortified in the livestock samples.

Chemical	Level	Recovery (%) *					
		Egg	Milk	Pork Bacon	Beef Steak	Beef Fat	Chicken Leg Meat
Fenpropimorph	LOQ	69.1 ± 2.6	75.5 ± 3.1	79.4 ± 2.9	92.4 ± 2.6	73.2 ± 2.6	91.3 ± 4.5
	2 × LOQ	76.7 ± 2.2	85.7 ± 2.9	78.5 ± 1.5	78.7 ± 3.3	82.2 ± 1.9	90.2 ± 3.3
	10 × LOQ	93.7 ± 1.7	82.6 ± 1.1	85.5 ± 1.5	83.7 ± 1.6	89.3 ± 0.7	100.6 ± 2.1
Fenpropimorph acid	LOQ	69.3 ± 2.7	72.1 ± 2.9	78.7 ± 1.8	97.0 ± 2.4	86.4 ± 4.1	111.1 ± 6.9
	2 × LOQ	76.6 ± 2.1	83.1 ± 1.6	76.3 ± 3.2	81.8 ± 1.7	91.3 ± 2.3	100.7 ± 5.6
	10 × LOQ	94.5 ± 2.2	70.1 ± 1.2	86.1 ± 1.7	84.7 ± 2.6	92.8 ± 0.7	111.2 ± 3.0

* Means ± SD of 5 replicates.

**Table 5:** Relative differences of the ion ratios of fenpropimorph and fenpropimorph acid in the livestock matrices.

Chemical	Level	Ion Ratio Difference (%) *					
		Egg	Milk	Pork Bacon	Beef Steak	Beef Fat	Chicken Leg Meat
Fenpropimorph	LOQ	−8.39	−5.36	3.48	−3.40	−5.65	−12.42
	2 × LOQ	−3.09	−6.13	3.64	−4.15	1.20	−8.19
	10 × LOQ	0.03	0.68	1.51	0.78	1.50	−7.60
Fenpropimorph acid	LOQ	−2.42	2.80	3.35	3.83	−4.35	−13.38
	2 × LOQ	−4.08	2.60	3.45	−0.77	−2.65	−5.53
	10 × LOQ	2.13	7.47	3.25	−0.51	−1.51	−8.24

* The relative difference in ion ratios (%) was calculated from the ion ratio of two MRM transitions in the sample and the ion ratio of two MRM transitions in the reference solvent, as described in the Materials and Methods section. The ion ratio in samples was averaged from five tests, and the ion ratio in reference solvents was averaged from the calibration solutions.

**Table 6:** Inter-institutional recovery values of fenpropimorph and fenpropimorph acid.

Chemical	Level	Inter-Institutional Recovery (%) *					
		Egg	Milk	Pork Bacon	Beef Steak	Beef Fat	Chicken Leg Meat
Fenpropimorph	LOQ	63.9 ± 2.5	75.1 ± 2.6	81.9 ± 2.1	70.3 ± 6.4	66.5 ± 1.3	85.9 ± 2.5
	2 × LOQ	76.5 ± 2.4	82.1 ± 3.2	80.4 ± 1.9	82.1 ± 4.3	76.1 ± 1.8	86.2 ± 2.9
	10 × LOQ	85.0 ± 0.3	89.6 ± 2.1	80.8 ± 1.9	81.1 ± 0.7	83.0 ± 0.8	80.7 ± 1.1
Fenpropimorph acid	LOQ	65.1 ± 3.2	73.9 ± 4.4	68.8 ± 4.8	66.1 ± 5.6	67.8 ± 2.8	63.1 ± 3.2
	2 × LOQ	74.7 ± 2.2	84.4 ± 3.6	77.4 ± 2.6	78.0 ± 5.9	80.0 ± 1.9	77.5 ± 2.8
	10 × LOQ	78.5 ± 1.0	95.9 ± 2.8	83.1 ± 1.3	83.5 ± 1.7	80.0 ± 1.2	83.9 ± 2.4

* Means ± SD of 5 replicates.

**Table 7:** Relative differences in the ion ratios of fenpropimorph and fenpropimorph acid in the real samples ^1^.

Chemical	Sample	Relative Difference of Ion Ratios (%) ^2^	
		Spiked Sample ^3^	Real Sample
Fenpropimorph	Egg	1.85	−98.33~630.66
	Milk	1.06	−93.58~82.82
	Pork bacon	−1.81	−92.35~110.36
	Beef steak	1.05	−64.02~2382.63
	Chicken leg meat	1.27	−58.67~196.49
Fenpropimorph acid	Egg	4.81	43.68~399.10
	Milk	−2.29	−80.82~−38.35
	Pork bacon	−1.80	−40.51~318.22
	Beef steak	−7.15	135.51~631.60
	Chicken leg meat	−1.99	243.40~2007.79

^1^ Means ± SD of 3 replicates. ^2^ The ratio range is between the markets. ^3^ Spiked at a rate of 0.01 µg/kg in each sample.

## Data Availability

Data are contained in the articles.

## Sample Availability

Samples of the compound are not available from the authors.
